# Prediction and Inverse Design of Structural Colors of Nanoparticle Systems via Deep Neural Network

**DOI:** 10.3390/nano11123339

**Published:** 2021-12-08

**Authors:** Lanxin Ma, Kaixiang Hu, Chengchao Wang, Jia-Yue Yang, Linhua Liu

**Affiliations:** 1 School of Energy and Power Engineering, Shandong University, Jinan 250061, China; malanxin@sdu.edu.cn (L.M.); kaixianghu@mail.sdu.edu.cn (K.H.); sduwcc18@sdu.edu.cn (C.W.); 2 Optics & Thermal Radiation Research Center, Institute of Frontier and Interdisciplinary Science, Shandong University, Qingdao 266237, China

**Keywords:** neural network, optical properties, nanoparticles, Mie scattering, Monte Carlo

## Abstract

Noniridescent and nonfading structural colors generated from metallic and dielectric nanoparticles with extraordinary optical properties hold great promise in applications such as image display, color printing, and information security. Yet, due to the strong wavelength dependence of optical constants and the radiation pattern, it is difficult and time-consuming to design nanoparticles with the desired hue, saturation, and brightness. Herein, we combined the Monte Carlo and Mie scattering simulations and a bidirectional neural network (BNN) to improve the design of gold nanoparticles’ structural colors. The optical simulations provided a dataset including color properties and geometric parameters of gold nanoparticle systems, while the BNN was proposed to accurately predict the structural colors of gold nanoparticle systems and inversely design the geometric parameters for the desired colors. Taking the human chromatic discrimination ability as a criterion, our proposed approach achieved a high accuracy of 99.83% on the predicted colors and 98.5% on the designed geometric parameters. This work provides a general method to accurately and efficiently design the structural colors of nanoparticle systems, which can be exploited in a variety of applications and contribute to the development of advanced optical materials.

## 1. Introduction

Color generation from the assembly of nanometer- or submicron-sized structures has been of great interest for various fields including chromatic displaying [1,2,3], color printing [4,5], and biomedical imaging [6]. By exploiting the light–matter interaction in the designed structures, the generated colors are superior to the traditional pigmentary colors in many ways, due to their large tunability, resistance to bleaching, and reduced dependence on toxic materials. Much recent research has demonstrated the use of ordered and disordered photonic structures to generate colors across the visible spectrum [7,8,9]. Periodically ordered photonic structures generate colors with the iridescent feature due to Bragg diffraction [10,11]. However, their colors are strongly dependent on the angle of view because of the periodic structure. In contrast, the structures consisting of metallic and dielectric nanoparticles can generate nonfading and noniridescent colors with high resolution, and have been most widely studied as a component to realize structural coloration [12,13,14,15,16,17].

The structural colors of nanoparticle systems depend on the material, size, shape, and volume fraction of nanoparticles, and the dielectric properties of the surrounding environment. Therefore, by adjusting the parameters as mentioned above, the corresponding spectra, either scattering, transmission, or reflection modified by nanoparticles, can be manipulated to obtain a variety of colors. However, the design of specific properties of nanoparticle systems is usually based on a trial-and-error method [18], which requires prior experiences and scientific intuitions but also a lot of time and labor costs. Many attempts have been made to improve the design efficiency via various optimization strategies such as using a particle swarm algorithm, topology optimization, and combining with GPU-acceleration [19,20,21]. However, traditional design and optimization methods are still facing challenges with the increasing complexity of system functionality and integration. Fortunately, machine learning methods based on artificial neural networks have emerged as a powerful tool in solving complex computation and inverse design problems. Machine learning is a statistics technology that trains a machine by telling it what to do, and has proven to be particularly good at solving the problems of classification and regression [22,23]. In the field of nanophotonics, machine learning has made great progress in many applications such as pattern recognition, optical imaging, and structure design [24,25,26,27]. Most recently, machine learning techniques have been utilized to inversely design the structure and material to achieve the desired optical and color properties [28,29,30,31,32,33]. Baxter et al. [28] proposed a deep learning method to predict the colors of silver surfaces doped with silver nanoparticles and to inversely design the geometric parameters for the desired colors. Gao et al. [29] trained a bidirectional deep neural network model to inversely design the structural color of silicon nanostructures. The model consists of a forward network and an inverse network, which can accurately predict the color generation of silicon nanostructures, as well as inversely design the structural parameters for the desired colors. Huang et al. [30] proposed an inverse design strategy for the structural color of dielectric ring arrays using a machine learning model that combines the supervised learning with reinforcement learning. Dai et al. [31] reported a bidirectional neural network to inversely design the geometric structures of Fabry–Perot cavity color filters, which enables a gamut coverage that is 215% of the sRGB color space.

The above studies have mainly focused on the periodic systems whose optical response is obtained using the time-consuming electromagnetic simulations; yet, disperse nanoparticle systems have received little attention. In this work, a multilayered BNN model was built to accurately predict the color generation of gold nanoparticle systems and to inversely design the geometric parameters for the desired colors. The single scattering properties of nanoparticles were calculated by using the Lorenz–Mie theory, the multiple scattering problems between nanoparticles were considered by solving the radiative transfer equation, and the spectrum-to-color conversion was obtained based on the International Commission on Illumination (CIE) color spaces. Our approach captures the complex relationships between the geometric parameters and structural colors with high reliability and accuracy, and can be used for the analysis and optimization design of structural color in nanoparticle systems.

## 2. Model and Methods

### 2.1. Model and Dataset Generation

**Figure 1:**
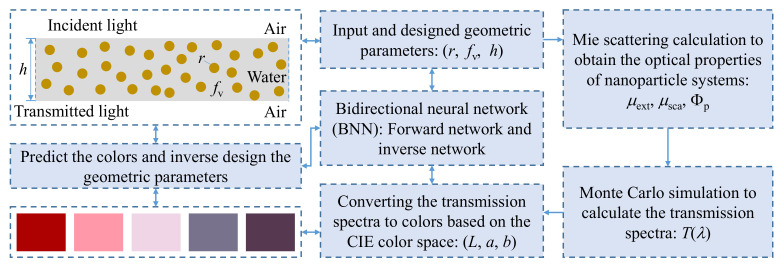
Schematic of the model and methods for the prediction and inverse design of structural colors of nanoparticle systems.

Our data generation process and machine learning strategy for structural colors of nanoparticle systems are schematically illustrated in Figure 1. A thin layer composed of randomly distributed, monodisperse spherical gold nanoparticles embedded in water was investigated, because of its strong plasmon resonances in the visible spectrum and wide application in nanophotonics [7,34,35,36]. The external medium was set to air. The dispersed layer can be considered as a typical model of colloidal suspensions, nanoparticles composite coatings, and thin-film materials. We selected three geometric parameters: particle radius *r*, particle volume fraction *f*_v_, and layer thickness *h*, and three color property parameters: (*L*, *a*, *b*) in the CIE 1976-Lab color space, as key features to establish the dataset for the prediction and inverse design of structural colors. The color property parameters of nanoparticle systems were obtained from a series of simulations, which include Mie scattering calculation, Monte Carlo simulation, and spectrum-to-color conversion. Then, a multilayered BNN was developed and trained to predict the structural colors of nanoparticle systems and inversely design the geometric parameters for the desired colors.

**Figure 2:**
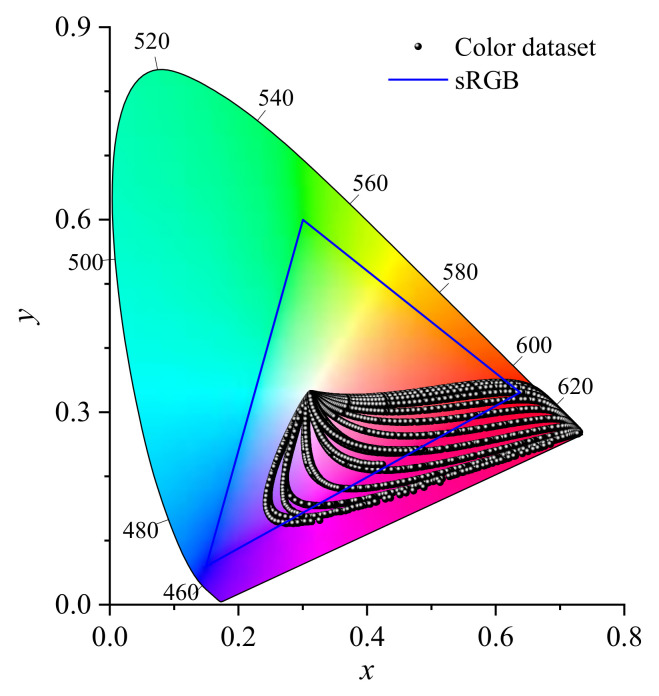
The 12,000 colors generated by gold nanoparticle systems plotted in a CIE 1931-XYZ chromaticity diagram. The boundary of the sRGB color space is marked with blue lines.

The range of *r* was set to 5–100 nm in 5 nm intervals, the range of *f*_v_ was set to 1 × 10^−6^–3 × 10^−5^ in 1 × 10^−6^ intervals, and the range of *h* was set to 0.5–10 mm in 0.5 mm intervals. A total of 12,000 parameter combinations were generated and the corresponding transmission spectra from 360 to 830 nm (in 1 nm intervals) were obtained by combining the Mie scattering and Monte Carlo calculation. Then, the color property parameters (*L*, *a*, *b*) were obtained by conducting the spectrum-to-color conversion. This dataset was divided into three groups for training (9600), validation (1200), and testing purposes (1200). All colors generated in the training and validation dataset are plotted in the CIE 1931-XYZ chromaticity diagram in Figure 2. More detailed information for the model and methods is provided in the following sections.

### 2.2. Optical Properties of Nanoparticle Systems

For dilute gold nanoparticles embedded in water, the total optical properties of nanoparticle systems, which include the extinction coefficients *μ*_ext_, scattering coefficients *μ*_sca_, and scattering phase functions Φ, are calculated as [37]:

(1) $$\mu_{\mathrm{ext}}=\mu_{\mathrm{ext},p}+\mu_{\mathrm{ext},m}$$

(2) $$\mu_{\mathrm{sca}}=\mu_{\mathrm{sca},p}$$

(3) $$\Phi =\Phi_{p}$$

where *μ*_ext,m_ *=* 4π*κ*/*λ* is the extinction coefficient of water, *κ* is the imaginary part of the refractive index of water, *λ* is the incident light wavelength, and *μ*_ext,p_, *μ*_sca,p_, and Φ_p_ are the extinction coefficient, scattering coefficient, and scattering phase function of gold nanoparticles, respectively. In the framework of the independent scattering approximation, the optical properties of dilute monodisperse gold nanoparticles can be calculated as [37]:

(4) $$\mu_{\mathrm{ext},p}=\frac{3}{4}Q_{\mathrm{ext},p}\frac{f_{v}}{r}$$

(5) $$\mu_{\mathrm{sca},p}=\frac{3}{4}Q_{\mathrm{sca},p}\frac{f_{v}}{r}$$

(6) $$\Phi_{p}=\Phi_{p,s}$$

where *Q*_ext,p_, *Q*_sca,p_, and Φ_p,s_ are the extinction efficiency factor, scattering efficiency factor, and scattering phase function of single gold spheres, respectively, and can be calculated using Lorenz–Mie theory [38]. The dielectric function of gold nanoparticles was taken from Johnson and Christy’s [39] dataset, and the complex refractive index of pure water [40] was used.

### 2.3. Calculation of Transmission Spectra

Given the optical properties of the nanoparticle systems, the radiative transfer problems (e.g., transmission and reflection spectra) can be accounted for by solving the radiative transfer equation (RTE) [37,41]:

(7) $$\frac{dI_{\lambda }\left(s\right)}{ds}=-\mu_{\mathrm{ext}}I_{\lambda }\left(s\right)+\left(\mu_{\mathrm{ext}}-\mu_{\mathrm{sca}}\right)I_{b\lambda }\left(s\right)+\frac{\mu_{\mathrm{sca}}}{4\pi }\int_{4\pi }^{}I_{\lambda }\left(s,\vec{\Omega }\prime \right)\Phi \left(\vec{\Omega }\prime ,\;\vec{\Omega }\prime \right)d\Omega \prime$$

where *I_λ_* is the spectral radiative intensity in the direction of $\vec{\Omega }$ along path *s*, *I_b__λ_* is the spectral blackbody intensity, and Ω′ is the solid angle. The RTE is solved using the Monte Carlo method [37,42,43]. An infinitely thin light beam is perpendicularly incident on the upper boundary of the layer from air, as shown in Figure 1. The reflection and refraction at the boundaries are considered using the Snell’s law and Fresnel’s relation [37]. After interacting with the layer, the reflected and transmitted photons are collected, and the spectral directional-hemispherical transmittance *T*(*λ*) of the layer can be calculated as:

(8) $$T\left(\lambda \right)=\frac{\sum_{2\pi }N_{t}\left(\Omega_{t}\right)}{N_{0}}$$

where *N*_0_ is the total number of the photons that are incident on the layer, and *N_t_*(Ω*_t_*) is the number of photons that are collected with the use of detectors positioned in the hemispherical space outside the lower surface in the solid angle Ω*_t_*. In this study, 10^6^ photons were used for each wavelength to obtain accurate results without using too much computation time.

### 2.4. Spectrum to Color Calculation

The colors perceived by the human eye result from the interplay between the sensitivity of the eye’s three cone cells, the spectral intensity of the light source, and the spectral reflection or transmission from the object. The CIE-1931-XYZ and CIE 1976-Lab color spaces were used to assess the generated colors of nanoparticle systems [44,45,46]. As the transmitted light has a wider coverage of color space compared to the reflected light, we only focused on the transmission spectra and colors of the gold nanoparticle systems for simplicity. In accordance with the proposals of the CIE-1931-XYZ, the tristimulus values *X*, *Y*, and *Z* are defined by [44,45,47]:

(9) $$X=100\frac{\int_{360\mathrm{nm}}^{830\mathrm{nm}}I_{D65}\left(\lambda \right)T\left(\lambda \right)\bar{x}\left(\lambda \right)d\lambda }{\int_{360\mathrm{nm}}^{830\mathrm{nm}}I_{D65}\left(\lambda \right)\bar{y}\left(\lambda \right)d\lambda }$$

(10) $$Y=100\frac{\int_{360\mathrm{nm}}^{830\mathrm{nm}}I_{D65}\left(\lambda \right)T\left(\lambda \right)\bar{y}\left(\lambda \right)d\lambda }{\int_{360\mathrm{nm}}^{830\mathrm{nm}}I_{D65}\left(\lambda \right)\bar{y}\left(\lambda \right)d\lambda }$$

(11) $$Z=100\frac{\int_{360\mathrm{nm}}^{830\mathrm{nm}}I_{D65}\left(\lambda \right)T\left(\lambda \right)\bar{z}\left(\lambda \right)d\lambda }{\int_{360\mathrm{nm}}^{830\mathrm{nm}}I_{D65}\left(\lambda \right)\bar{y}\left(\lambda \right)d\lambda }$$

where *I*_D65_(*λ*) is the spectral power distribution of the standard D_65_ illumination. $\bar{x}\left(\lambda \right)$, $\bar{y}\left(\lambda \right)$, and $\bar{z}\left(\lambda \right)$ are the spectral tristimulus values, which contain information about the used light source [44]. The chromaticity of the color is obtained by the normalized values:

(12) $$x=\frac{X}{X+Y+Z}$$

(13) $$y=\frac{Y}{X+Y+Z}$$

(14) $$\;z=\frac{Z}{X+Y+Z}$$

The normalized values *x* and *y* are used to determine the corresponding chromaticity in CIE-1931-XYZ color space. In addition, the CIE 1976-Lab has a one-to-one correspondence to the CIE-1931-XYZ but with much better uniformity, rendering it a more suitable color space for accurate color difference identification [31,46]. The CIE 1976-Lab color space is defined in terms of the tristimulus values *L*, *a*, and *b*, where *L* represents the lightness, *a* represents the redness (+) and greenness (−), and *b* represents the yellowness (+) and blueness (−). The conversion relation between (*X*, *Y*, *Z*) and (*L*, *a*, *b*) is stated as [46]:

(15) $$L=116f\left(Y/Y_{n}\right)-16$$

(16) $$a=500\left[f\left(X/X_{n}\right)-f\left(Y/Y_{n}\right)\right]$$

(17) $$b=200\left[f\left(Y/Y_{n}\right)-f\left(Z/Z_{n}\right)\right]$$

with *X_n_*, *Y_n_*, and *Z_n_* being the tristimulus values of a reference white object, and:

(18) $$f\left(s\right)=s^{1/3}\;\mathrm{if}\;s>{\left(24/116\right)}^{3}$$

(19) $$\;f\left(s\right)=\left(841/108\right)s+16/116\;\mathrm{if}\;s<{\left(24/116\right)}^{3}$$

For the standard illuminant D_65_ and a 2^°^ standard colorimetric observer, they amount to *X_n_* = 95.049, *Y_n_* = 100, and *Z_n_* = 108.891. In contrast to *X*, *Y*, and *Z*, the tristimulus values *L*, *a*, and *b* allow the quantitative comparison of colors. The CIE color difference function Δ*E* can be defined as the Euclidean distance between two vectors (*L*, *a*, *b*) and (*L*′, *a*′, *b*′) [46,48].

(20) $$\Delta E=\sqrt{{\left(L\prime -L\right)}^{2}+{\left(a\prime -a\right)}^{2}+{\left(b\prime -b\right)}^{2}}$$

and a value of Δ*E* ≤ 1.0 usually represents identical colors [14,48].

### 2.5. Machine Learning Method

**Figure 3:**
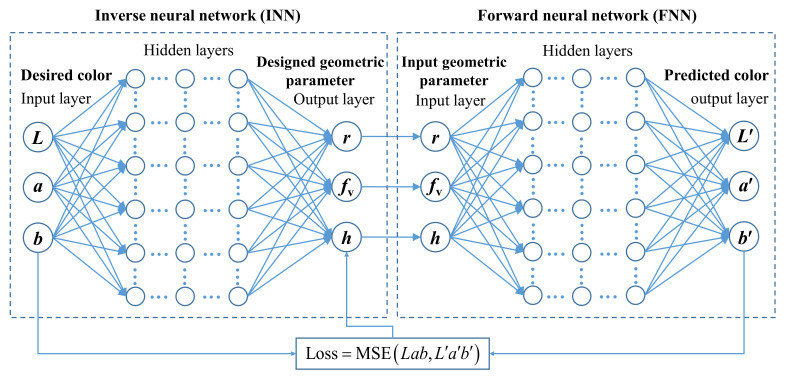
Simplified schematic of the multilayered BNN model that consists of a fully connected INN that directly connects to a pre-trained FNN.

A multilayered BNN model, first proposed by Liu [49], was utilized to predict the color generation of the gold nanoparticle systems and inverse design of geometric parameters for the desired colors. The model consists of a fully connected inverse neural network (INN) that directly connects to a pre-trained forward neural network (FNN), as shown in Figure 3. The major advantage of this model is the excellent ability to solve the so-called “one-to-many” problem, which describes the fact that most inverse design problems are ambiguous, and hence several non-unique solutions exist for the same design target [21,49].

Prior to training, the geometric properties of nanoparticle systems and the corresponding color properties are normalized to improve the convergence rate and reduce the training error. In the training process, the FNN is first trained to achieve accurate prediction of the structural colors (*L*, *a*, *b*) based on the input geometric parameters (*r*, *f*_v_, *h*). The number of layers and neurons in the hidden layer are determined by neural network training and construction, and the training is performed by minimizing the loss function, which is defined as the mean square error (MSE) between the predicted values ($L_{\mathrm{predicted}}^{\prime }$, $a_{\mathrm{predicted}}^{\prime }$, $b_{\mathrm{predicted}}^{\prime }$) and true values (*L*_true_, *a*_true_, *b*_true_):

(21) $${\mathrm{MSE}}_{Lab}=\frac{1}{N}\overset{N}{\underset{i=1}{\sum }}\left[{\left(L_{\mathrm{predicted}}^{\prime }-L_{\mathrm{true}}\right)}^{2}+{\left(a_{\mathrm{predicted}}^{\prime }-a_{\mathrm{true}}\right)}^{2}\;+{\left(b_{\mathrm{predicted}}^{\prime }-b_{\mathrm{true}}\right)}^{2}\right]$$

where *N* is the number of the entries of color data. The training of the INN subsequently uses the fixed pre-trained FNN model as a color predictor to evaluate the inverse design output. Note that the loss function of INN compares the output color properties (*L*, *a*, *b*) rather than the designed geometric parameters (*r*, *f*_v_, *h*), as shown in Figure 3. In this way, different geometric parameters that lead to a similar color response no longer confuse the neural network, and all correct solutions to a given design problem yield a positive training feedback.

## 3. Results and Discussion

### 3.1. Structural Colors of Gold Nanoparticle Systems

Due to the particle scattering and absorption, the colors of the dispersed layer are determined by various factors such as the size, shape, volume fraction, and refractive index of nanoparticles, and the thickness and surface condition of the layer. In this study, we focused on the influence of particle size, volume fraction, and layer thickness on the structural colors of gold nanoparticle systems. As shown in Figure 2, a relatively wide coverage of the CIE color space is observed, and the transmission light mainly appears as purple and red colors, some of which are even out of the range of the sRGB color space. These results confirm that a wide range of structural colors can be achieved by using only one type of nanoparticle through controlling the specific geometric parameters.

**Figure 4:**
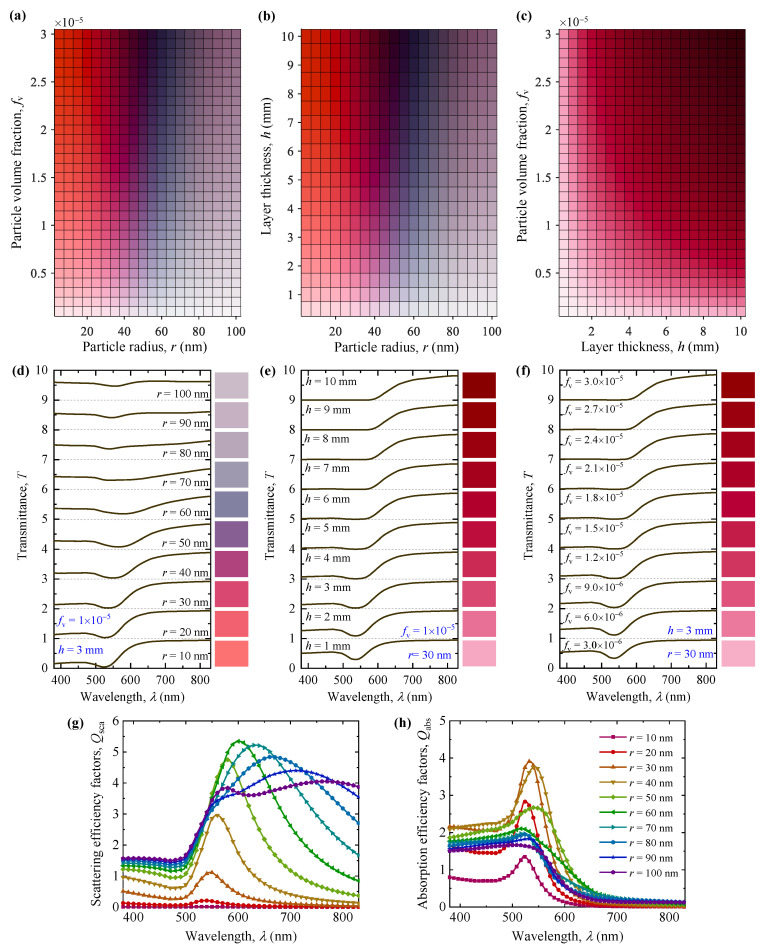
The generated colors of gold nanoparticle systems for different geometric parameters with (**a**) *h* = 3.0 mm, (**b**) *f*_v_ = 1 × 10^−5^, and (**c**) *r* = 30 nm. The simulated transmittance spectra of gold nanoparticle systems with (**d**) *h* = 3.0 mm, *f*_v_ = 1 × 10^−5^, and different particle radii; (**e**) *f*_v_ = 1 × 10^−5^, *r* = 30 nm, and different layer thicknesses; and (**f**) *r* = 30 nm, *h* = 3.0 mm, and different particle volume fractions. Note that the curves in (**d**–**f**) with different geometric parameters are successively displaced upward by 1 for better visibility and comparison. (**g**) The scattering and (**h**) absorption efficiency factors of single particles for different particle radii.

The influences of different geometric parameters on the generated colors and transmission spectra of gold nanoparticle systems are presented in Figure 4. Figure 4a–c shows the generated colors for different particle radii, particle volume fractions, and layer thicknesses. Figure 4d–f shows the simulated transmittance spectra of gold nanoparticle systems. To better illustrate the change in color chromaticity, Table S1 lists the corresponding color property parameters *L*, *a*, and *b* of the generated colors in Figure 4d–f. For the sake of comparing and analyzing, the scattering and absorption efficiency factors of single particles for different particle radii are also illustrated in Figure 4g,h. As shown, the particle radius, particle volume fraction, and layer thickness have different degrees of influence on the generated colors and transmission spectra. Compared with the impacts of particle volume fraction and layer thickness, the particle radius shows a greater impact on the color chromaticity. With increasing particle radius, the values of *a* and *b* decrease first and then increase, resulting in the colors of the gold nanoparticle system changing from red to purple, then grey, and then light-grey, as shown in Figure 4a,b,d and Table S1. For small-size particles with particle radii smaller than 30 nm, the transmitted light mainly appears as red and pink colors. This is mainly due to the strong absorption and weak scattering of light in the blue and green region, which results in the red light passing through the layers with high transmittance, as illustrated in Figure 4d,g,h. As the particle radius increases, the shifts in the resonance peaks and enhanced scattering effect result in different distribution patterns of transmittance spectra, and further lead to obvious changes in the generated colors.

In addition, we can observe that the particle volume fraction and layer thickness mainly impact the lightness *L* of the nanoparticle systems, as shown in Figure 4a–c and Table S1. For nanoparticle systems with low particle volume fraction and layer thickness, due to the weak extinction of gold nanoparticles, different wavelengths of transmitted light are mixed together and exhibit the white and pink colors with high lightness. As the particle volume fraction and layer thickness increase, the multiple scattering effects between gold nanoparticles obviously increase, which not only result in the enhancement of particle extinction and the decrease in transmittance and lightness, but also impact the generated colors in different degrees.

On the whole, by adjusting the geometric parameters of nanoparticle systems and controlling the multiple scattering between nanoparticles, a wide range of structural colors can be obtained. Meanwhile, it should be noted that the effects of particle size distribution, particle shape, and particle aggregation were not considered in this study, which also have important influences on the structural colors of nanoparticle systems [14,15,43]. These influence factors will be investigated in future work.

### 3.2. Forward Neural Networks to Predict the Color Generation

**Figure 5:**
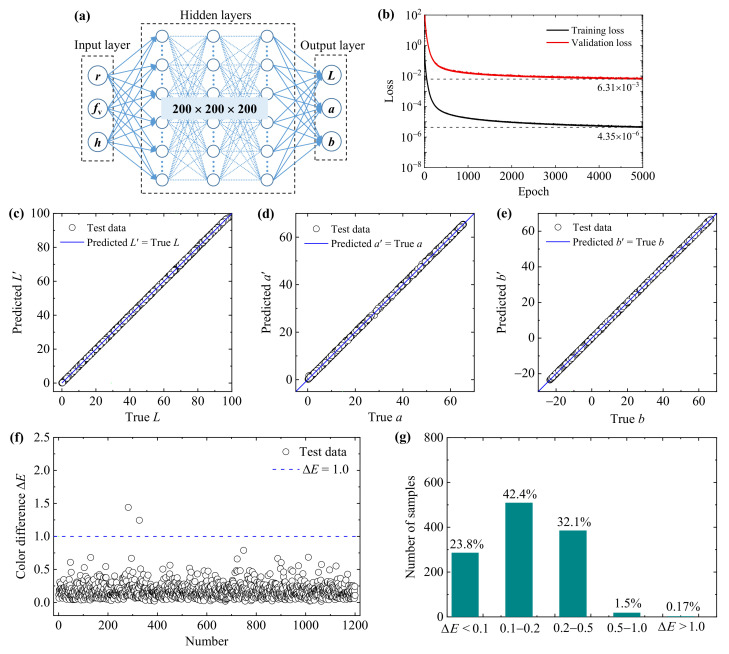
The forward neural network for predicting the colors of gold nanoparticle systems. (**a**) Structure of the forward neural network. (**b**) The loss functions of the training and validation sets. (**c**–**e**) Scatter plot of the true colors (*L*, *a*, *b*) and predicted colors (*L*′, *a*′, *b*′) for the 1200 groups of test data. (**f**) The color differences Δ*E* between the predicted colors and true colors. (**g**) Statistical distributions of the testing results divided by different ranges of color differences.

A fully connected FNN, which consists of one input layer, several hidden layers, and one output layer, was trained to accurately predict the structural colors of nanoparticle systems. Notably, 9600 groups of geometric parameters in the training set were fed to the FNN, which predicts the color properties, calculates the loss function, and updates the parameters such as weights and biases according to the back-propagation of loss function. In addition, 1200 groups of validation data were used to adjust the hyper-parameters and check the performance of the neural network during training. By minimizing the training and validation loss, and through continuously optimizing the structure parameters, a FNN structure with 3 hidden layers and 200 nodes per layer shows excellent performance with high accuracy, as shown in Figure 5a. The loss functions of training and validation sets over epochs are plotted in Figure 5b. As shown, the loss function decreases rapidly at the beginning of the training process due to a large gradient, and then the downward trend slows gradually. After 5000 epochs, the values of training and validation loss are 4.35 × 10^−6^ and 6.31 × 10^−3^, respectively. The color difference Δ*E* corresponding to the validation loss 6.31 × 10^−3^ is 0.079, which is difficult to recognize by the human eye. The smooth downward trend and the small loss values show that the FNN performs well in the training process.

In order to test the accuracy and generalization ability of the FNN, 1200 groups of completely new test data were used and analyzed. Figure 5c–e shows the comparison results between the predicted color property parameters (*L*′, *a*′, *b*′) and true values (*L*, *a*, *b*). The results obtained by the FNN show a high consistency with those obtained by the theoretical simulations, which demonstrates that the FNN has a good prediction ability for the sample of the test set. To better evaluate the performance of the FNN, the color differences Δ*E* between the predicted colors and true colors and their statistical distributions are illustrated in Figure 5f,g. In general, the human eye cannot identify the color difference Δ*E* < 1.0 [14,48]. As shown in the figure, only two of the color difference values (0.17%) are larger than 1.0, which means that the accuracy of FNN is 99.83% according to the human chromatic discrimination ability. Meanwhile, most of the color difference values (98.33%) are below 0.5, proving that the prediction of structural colors of nanoparticle systems based on the FNN is highly accurate.

**Figure 6:**
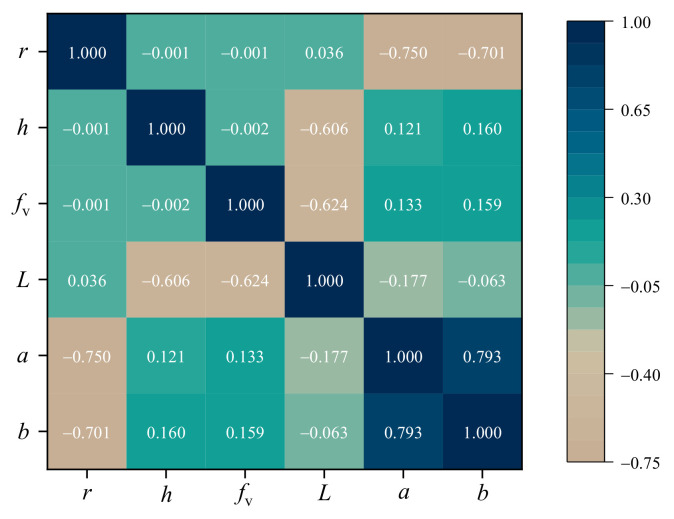
The heat map of the Pearson correlation coefficient matrix within the input geometric parameters (*r*, *f*_v_, *h*) and output color properties (*L*, *a*, *b*).

To further investigate the relationship between the geometric parameters and color properties, a heat map of the Pearson correlation coefficient matrix is illustrated in Figure 6. The Pearson correlation coefficient is a measure of the linear association between two features and has a value between −1 and 1. Positive values indicate positive correlations, while negative values indicate negative correlations. The further away the correlation coefficient is from zero, the stronger the relationship between the two features becomes. As shown in Figure 6, the correlation coefficients between the particle radius r and the color property parameters *L*, *a*, and *b* are 0.036, −0.75, and −0.701, respectively, which indicates that the particle radius shows strong negative correlation with the chromaticity and has weak correlation with the lightness. On the contrary, the layer thickness and particle volume fraction show strong negative correlation with the lightness and weak positive correlation with the chromaticity. These results are consistent with the theoretical analysis in Section 3.1, which are mainly caused by the single scattering and multiple scattering of gold nanoparticles, and verify that our FNN can accurately capture the complex relationships between the geometric parameters and structural colors.

Overall, the application of FNN can perform the accurate prediction of structural colors of nanoparticle systems with high reliability and efficiency. Although the acquisition of 12,000 groups of training data requires a certain computational cost, after the initial one-time computation investment, the FNN method has a great advantage in the computational efficiency and can be easily and flexibly used in the related applications.

### 3.3. Inverse Neural Networks to Design the Geometric Parameters

**Figure 7:**
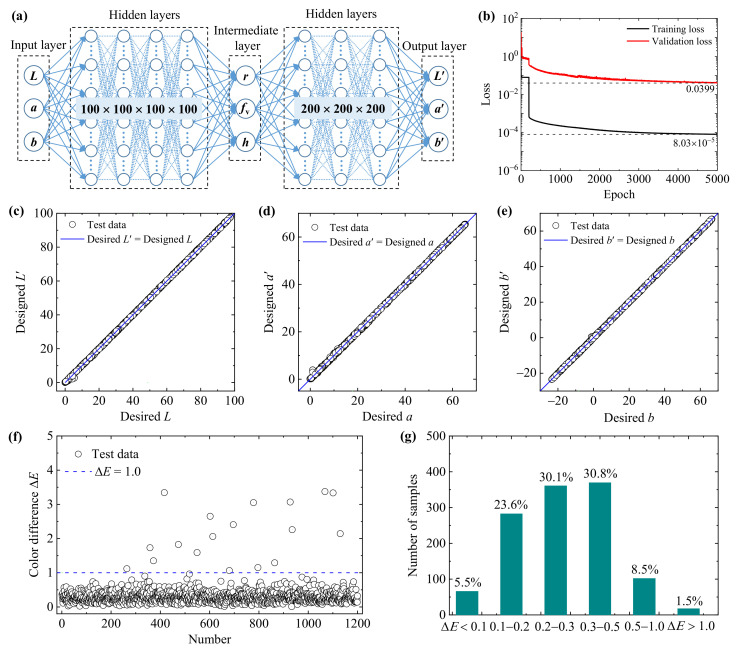
The bidirectional neural network for designing the geometric parameters of gold nanoparticle systems. (**a**) Structure of the multilayered BNN. (**b**) The loss functions of the training and validation sets. (**c**–**e**) Scatter plot of the desired colors (*L*, *a*, *b*) and predicted colors (*L*′, *a*′, *b*′) for the 1200 groups of test data. (**f**) The color differences Δ*E* between the designed colors and desired colors. (**g**) Statistical distributions of the testing results divided by different ranges of color differences.

Inverse design is much more complicated because different geometric parameters of nanoparticle systems may yield the same color, and “one-to-many” results may lead to untrained models. Introducing a bidirectional neural network with a tandem strategy is an effective way to solve the “one-to-many” problem [18,49]. As shown in Figure 3, the INN is directly connected to the pre-trained FNN. After completing the training of the FNN, the weights in the FNN are fixed and then the training of the INN is carried out. In the training process of the INN, the desired colors (*L*, *a*, *b*) are taken as the input, and the output is the designed geometric parameters (*r*, *f*_v_, *h*) denoted as the intermediate layer. The output of the pre-trained FNN is the predicted colors (*L*′, *a*′, *b*′) obtained based on the designed geometric parameters (*r*, *f*_v_, *h*). The weights in the INN are optimized by minimizing the loss function defined as the mean square error between the output predicted colors (*L*′, *a*′, *b*′) and the input desired colors (*L*, *a*, *b*). After optimization design, an INN model, which has 4 hidden layers with 100 neurons each layer, is constructed, as shown in Figure 7a. The training loss and validation loss curves of the INN are plotted in Figure 7b.

As shown in Figure 7b, the loss functions decrease rapidly at the beginning of the training process and then remain stable until about the 200^th^ epoch. After a sudden drop, the downward trend of the loss function gradually slows, and the values of training and validation loss are 8.03 × 10^−5^ and 0.0399, respectively, at the 5000^th^ epoch. Note that the value of validation loss 0.0399 corresponds to the color difference Δ*E* = 0.1997, showing that the INN performs well in the training process. We again use 1200 groups of new data to test the accuracy and generalization ability of the INN. The desired color properties (*L*, *a*, *b*) are input into the INN to predict the designed geometric parameters (*r*, *f*_v_, *h*). Then, the data of (*r*, *f*_v_, *h*) are fed to the FNN to obtain the designed color properties (*L*′, *a*′, *b*′). Figure 7c–g illustrates the comparison results between the desired color properties (*L*, *a*, *b*) and inversely designed color properties (*L*′, *a*′, *b*′), and the corresponding results of color differences Δ*E*. Good consistency between the desired and inversely designed color properties is observed. Most of the color difference values (90%) are below 0.5, and only 1.5% of the values are larger than 1.0, indicating that the inverse design of structural colors of nanoparticle systems based on the INN is highly accurate (with a high accuracy of 98.5%).

**Figure 8:**
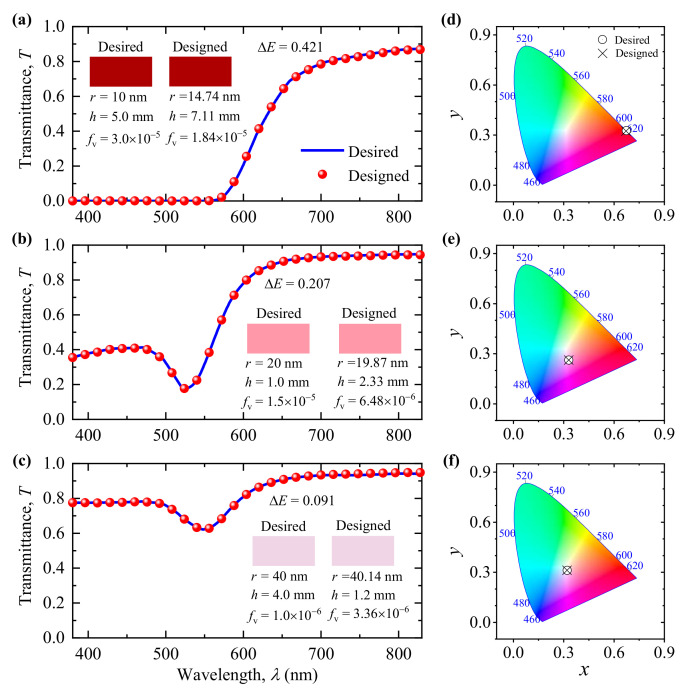
Inversely designing the geometric parameters of gold nanoparticle systems for desired colors. (**a**–**c**) The transmittance spectra of the desired (lines) and designed (points) colors. (**d**–**f**) The corresponding CIE chromaticity coordinates of the desired (circles) and designed (crosses) colors. The geometric parameters for both desired and designed colors and the corresponding color differences are inserted in (**a**–**c**). The color property parameters are as follows: (**a**) Desired color: *L* = 34.09, *a* = 63.99, *b* = 58.25; designed color: *L* = 34.49, *a* = 64.04, *b* = 58.37. (**b**) Desired color: *L* = 73.96, *a* = 40.44, *b* = 8.03; designed color: *L* = 73.88, *a* = 40.57, *b* = 8.17. (**c**) Desired color: *L* = 87.82, *a* = 11.78, *b* = −4.79; designed color: *L* = 87.75, *a* = 11.81, *b* = −4.84.

Furthermore, we randomly used three groups of data in the test set to evaluate the performance of the INN. Figure 8a–c shows transmittance spectra of the desired and designed colors. Note that the geometric parameters for both desired and designed colors and the corresponding color differences are inserted in the figure. Figure 8d–f shows the corresponding CIE chromaticity coordinates of the desired and designed colors. Moreover, for convenience of contrast, the reflectance and absorptance spectra of the desired and designed colors are shown in Figure S1. As shown in Figure 8 and Figure S1, the designed colors and the corresponding transmittance, reflectance, and absorptance spectra show excellent agreement with the results of desired colors. Moreover, we observe that the designed geometric parameters obtained based on the INN are different from the input geometric parameters for all three groups of data, although the color differences Δ*E* between the desired and designed colors are very small, as shown in Figure 8a–c. This result not only verifies that the nanoparticle systems composed of different geometric parameters can yield the same or similar colors, but also demonstrates the ability of the INN to discover new solutions outside of the training data. Overall, after the one-time investment of sufficient simulation data and the training of the BNN model, the prediction and inverse design of colors take only a few seconds, which can greatly reduce the cost and time of the colors’ design.

To summarize, our inverse design strategy based on a BNN bypasses the complicated simulations in searching for geometric parameters of nanoparticle systems for the desired colors, and so greatly accelerates the design of structural colors with high accuracy and efficiency. Our work provides a general method to accurately and efficiently design the structural colors of nanoparticle systems. Meanwhile, our model can be further extended to more complex nanoparticle systems by using additional information such as particle type, shape, and size distribution.

## 4. Conclusions

In summary, we combined the Monte Carlo, Mie scattering simulations, and bidirectional neural network model to successfully improve the design of structural colors in gold nanoparticle systems. A FNN model with 3 hidden layers including 200 neurons each layer, and an INN model of 4 hidden layers containing 100 neurons each layer were constructed. The proposed neural network could accurately capture the complex relationships between the geometric parameters and structural colors. Taking the human chromatic discrimination ability as a criterion, our proposed neural network achieved a high accuracy of 99.83% on the predicted colors and 98.5% on the designed geometric parameters. Our work demonstrates the applicability of the deep learning method for the prediction and inverse design of structural colors in nanoparticle systems, and will open a new way for the further exploration of the related applications about nanoparticle-based materials.

## Data Availability

The data presented in this study are available on request from the corresponding author.

## Supplementary Materials

The following are available online at www.mdpi.com/article/10.3390/nano11123339/s1, Figure S1. Inversely designing the geometric parameters of gold nanoparticle systems for desired colors. (a–c) The reflectance spectra of the desired (lines) and designed (points) colors. (d–f) The absorptance spectra of the desired (lines) and designed (points) colors, Table S1. Color properties of gold nanoparticle systems composed of different geometric parameters.
