# Supplementary material for: Prediction and Inverse Design of Structural Colors of Nanoparticle Systems via Deep Neural Network
Source: Nanomaterials (Basel). 2021 Dec 8;11(12):3339. doi: 10.3390/nano11123339 (PMC8703294; doi:10.3390/nano11123339)
Supplement: Supplementary file 1 [file nanomaterials-11-03339-s001.zip › nanomaterials-1483557-supplementary.pdf]

nanomaterials

MDPI

Supplementary Materials

Prediction and Inverse Design of Structural Colors of Nanoparticle Systems via Deep Neural Network

Lanxin Ma [1,2], Kaixiang Hu [1,2], Chengchao Wang [1,2], Jia-Yue Yang [1,2,*] and Linhua Liu [1,2,*]

1 School of Energy and Power Engineering, Shandong University, Jinan 250061, China; malanxin@sdu.edu.cn (L.M.); kaixianghu@mail.sdu.edu.cn (K.H.); sduwcc18@sdu.edu.cn (C.W.)
2 Optics & Thermal Radiation Research Center, Institute of Frontier and Interdisciplinary Science, Shandong University, Qingdao 266237, China
* Correspondence: jy_yang@sdu.edu.cn (J.-Y.Y.); liulinhua@sdu.edu.cn (L.L.)

Abstract: Noniridescent and nonfading structural colors generated from metallic and dielectric nanoparticles with extraordinary optical properties hold great promise in applications such as image display, color printing and information security. Yet, due to the strong wavelength dependence of optical constants and radiation pattern, it is difficult and time-consuming to design nanoparticles with the desired hue, saturation and brightness. Herein, we combine the Monte Carlo and Mie scattering simulations and a bidirectional neural network (BNN) to improve the design of gold nanoparticles' structural colors. The optical simulations provide dataset including color properties and geometric parameters of gold nanoparticle systems, while the BNN is proposed to accurately predict the structural colors of gold nanoparticle systems and inversely design the geometric parameters for desired colors. Taking the human chromatic discrimination ability as a criterion, our proposed approach achieves a high accuracy of 99.83% on the predicted colors and 98.5% on the designed geometric parameters. This work provides a general method to accurately and efficiently design the structural colors of nanoparticle systems, which can be exploited in a variety of applications and contribute to the development of advanced optical materials.

Keywords: neural network; optical properties; nanoparticles; Mie scattering; Monte Carlo

Table S1. Color properties of gold nanoparticle systems composed of different geometric parameters.

h = 3 mm, $f_v = 1.0 \times 10^{-5}$				r = 30 mm, $f_v = 1.0 \times 10^{-5}$				r = 30 nm, h = 3 mm,			
r (nm)	L	a	b	h (mm)	L	a	b	f_v	L	a	b
10	64.93	52.65	25.63	1.0	76.73	32.79	−1.97	3.0×10^{-6}	78.52	30.26	−2.13
20	60.29	56.83	21.59	2.0	62.27	50.78	2.02	6.0×10^{-6}	64.71	48.08	0.90
30	52.43	59.54	8.76	3.0	52.43	59.54	8.76	9.0×10^{-6}	55.01	57.60	6.61
40	45.11	51.00	−11.00	4.0	45.44	63.11	16.15	1.2×10^{-5}	47.97	62.11	13.21
50	46.15	26.13	−21.48	5.0	40.20	64.01	23.11	1.5×10^{-5}	42.65	63.79	19.78
60	55.24	7.91	−17.41	6.0	36.07	63.56	29.13	1.8×10^{-5}	38.45	63.95	25.63
70	64.32	5.76	−10.33	7.0	32.68	62.49	34.03	2.1×10^{-5}	35.00	63.31	30.73
80	70.31	8.60	−6.57	8.0	29.81	61.09	37.93	2.4×10^{-5}	32.09	62.25	34.95
90	74.15	10.04	−5.57	9.0	27.32	59.56	39.62	2.7×10^{-5}	29.57	60.98	38.23
100	77.20	8.57	−5.17	10.0	25.11	57.98	39.12	3.0×10^{-5}	27.35	59.61	39.67

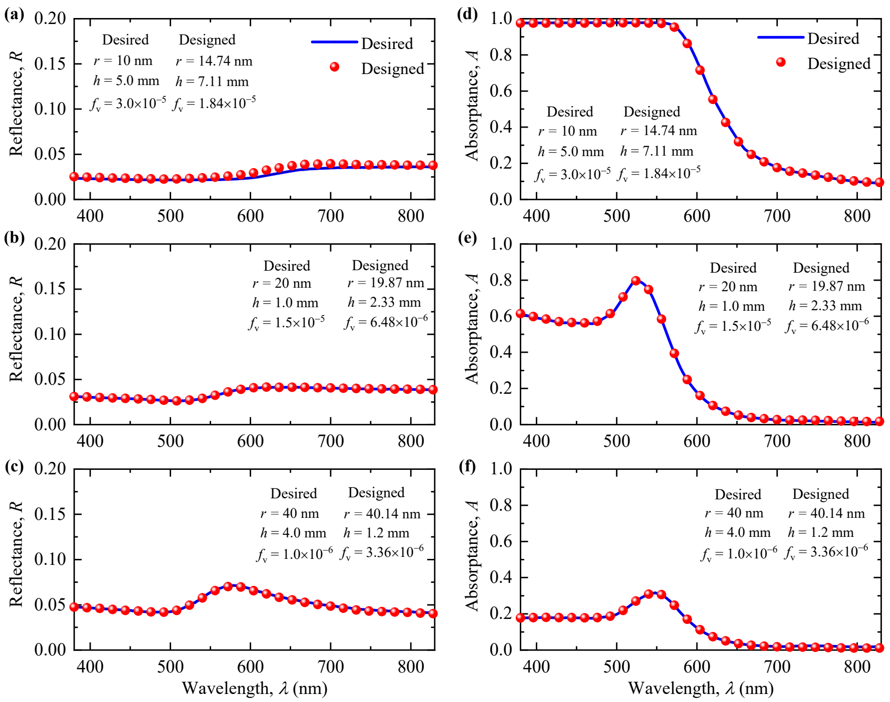

Figure S1. Inverse designing the geometric parameters of gold nanoparticle systems for desired colors. (**a**–**c**) The reflectance spectra of the desired (lines) and designed (points) colors. (**d**–**f**) The absorptance spectra of the desired (lines) and designed (points) colors. The color property parameters are as follows: (**a**,**d**) Desired color: L = 34.09, a = 63.99, b = 58.25; Designed color: L = 34.49, a = 64.04, b = 58.37. (**b**,**e**) Desired color: L = 73.96, a = 40.44, b = 8.03; Designed color: L = 73.88, a = 40.57, b = 8.17. (**c**,**f**) Desired color: L = 87.82, a = 11.78, b = −4.79; Designed color: L = 87.75, a = 11.81, b = −4.84.
